# Air Pollution during Pregnancy and Childhood Autism Spectrum Disorder in Taiwan

**DOI:** 10.3390/ijerph18189784

**Published:** 2021-09-17

**Authors:** Shu-Yuan Wang, Ya-Yun Cheng, How-Ran Guo, Yen-Cheng Tseng

**Affiliations:** 1 Department of Environmental and Occupational Health, College of Medical, National Cheng Kung University, Tainan 704, Taiwan; peter27312009@gmail.com (S.-Y.W.); b507092063@tmu.edu.tw (Y.-Y.C.); hrguo@mail.ncku.edu.tw (H.-R.G.); 2 Department of Environmental Health, Harvard University T.H. Chan School of Public Health, Boston, MA 02115, USA; 3 Department of Occupational and Environmental Medicine, National Cheng Kung University Hospital, Tainan 704, Taiwan; 4 Department of Tourism, Food, and Beverage Management, College of Management, Chang Jung Christian University, Tainan 711, Taiwan

**Keywords:** air pollution, autism spectrum disorder, carbon monoxide, nitrogen dioxide, particulate matters, gestation

## Abstract

Air pollutants have been linked to some diseases in humans, but their effects on the nervous system were less frequently evaluated. Autism spectrum disorder (ASD) is a group of neurondevelopmental disorders of which the etiology is still unknown. We conducted a study in Taiwan to evaluate the possible associations between prenatal exposure to air pollutants and ASD. From a random sample of one million people in the National Insurance Research Database, we identified all the infants born between 1996 and 2000. We followed them till the end of 2013 and identified cases of ASD. We traced back the mothers’ residence and assessed the exposure to air pollutants using the data obtained from the air quality monitoring database maintained by the government, which included ozone (O_3_), carbon monoxide (CO), nitrogen dioxide (NO_2_), sulfur dioxide (SO_2_), and particulate matters with diameter less than 10 µm (PM_10_). Cox proportional hazard models were constructed to evaluate the associations between childhood ASD and exposures to the pollutants in the three trimesters and the whole gestation. We identified a total of 63,376 newborns and included 62,919 as the study cohort. After adjusting for other risk factors, we observed trimester-specific associations between levels of CO, NO_2_, and PM_10_ and the risk of childhood ASD. An increase of 1 ppm of CO in the first, second, and third trimester was associated with a hazard ratio (HR) of 1.93 (95% confidence interval [CI]: 1.55–2.39), 1.77 (95%CI: 1.41–2.22), and 1.75 (95%CI: 1.39–2.21), respectively. An increase of 10 ppb in the level of NO_2_ in the first, second, and third trimester was associated with an HR of 1.39 (95%CI: 1.22–1.58), 1.25 (95%CI: 1.10–1.42), and 1.18 (95%CI: 1.03–1.34), respectively. In conclusion, we found that exposures to CO and NO_2_ in all three trimesters were associated with increased risks of developing ASD.

## 1. Introduction

Autism spectrum disorder (ASD) is a group of neurodevelopmental disorders that contains autistic disorder, Asperger’s disorder, and pervasive developmental disorder not otherwise specified (PDD-NOS) [1]. ASD was once regarded as a rare disease, and a study in the 1960s showed that the prevalence was 4.5 cases per 10,000 children [2]. However, the prevalence of ASD has been increasing dramatically since 1990s [3,4]. Reviews of the literature showed that it was 4.7 cases per 10,000 children in 1990–1993 but had rapidly increased to 12.7 cases per 10,000 children in 1994–2004 [5,6]. In the latest decade, the prevalence has reached 63.5 cases per 10,000 children [7]. There is also an increasing trend in Asia. Studies in Asia reported a prevalence of nearly 2 cases per 10,000 children in the 1970s [8,9,10,11], and the prevalence became over 10 cases per 10,000 children about four decades later [12]. A meta-analysis of studies in Asian countries found the prevalence elevated annually, from 8.5 cases per 10,000 children in 2000–2004 to 10.3 per 10,000 in 2005–2009, and then 16.4 per 10,000 children in 2011 [13]. Taiwan is no exception. An analysis of data from the national health insurance found the prevalence increased from 1.79 to 28.72 cases per 10,000 children between 1997 and 2005 [14], and an analysis of data from the national disability registry found disabling ASD increased from 3.7 to 22.1 cases per 10,000 children between 2004 and 2010 [15]. This increase might represent the changing in diagnostic tools, case definitions, awareness of the public, and health service accessibility [5,12,16]. However, a true increase in the incidence rate [17,18,19] might also contribute to the increasing prevalence [1,5,6,20,21].

The main characteristics of ASD are abnormal development or impairment in social interaction, impaired verbal and nonverbal communication, and markedly restricted activities and interests (American Psychiatric Association 2000). The cause of ASD is still unknown so far. It was once regarded as a genetic disease [3,22]. However, recent twin studies reported that both genetic and environmental factors contribute to the disease, and environmental factors might even contribute as high as 55% of the cause [23,24]. Air pollution during pregnancy has been known as an environmental factor for adverse pregnant health outcomes. High levels of air pollutants during pregnancy, including carbon monoxide (CO), ozone (O_3_), polycyclic organic matter, and particulate matters less than 10 μm in diameter (PM_10_), were found to be associated with low birth weight, preterm birth, and stillbirth [25,26,27]. Physical differences, including a reduction in biparietal diameter and head circumference, have also been observed in children born to mothers with exposure to air pollutants during pregnancy, including PM_10_, O_3_, nitrogen dioxide (NO_2_), and sulfur dioxide (SO_2_) [28]. Furthermore, exposure to air pollution was found to be associated with developmental outcomes. Infants with exposure to high levels of black carbon and NO_2_ were found to have decreased cognitive and intelligence performance [29,30]. Therefore, air pollution is a plausible environmental factor for ASD, and the rapidly increasing levels of air pollutants might contribute to the fast growing number of patients of ASD globally.

In fact, there is growing literature on the associations between ambient air pollution and ASD [31,32,33,34,35,36]. Many of the studies were case-control studies, which often suffer from information bias in the reporting of health outcomes. Although some studies found associations between air pollution and ASD, the exposure periods studied varied across studies, and findings on the critical exposure period were inconsistent. Besides, studies on Asian populations are still limited. Therefore, we used the Taiwan National Health Insurance Research Database and Taiwan air monitoring database to conduct a retrospective cohort study, which evaluated the associations between the ambient air pollution exposure in different gestation periods and the risk of ASD.

## 2. Materials and Methods

### 2.1. Study Cohort

This population-based cohort study included members from the Longitudinal Health Insurance Database 2000 (LHID2000), which randomly sampled one million individuals who were covered by the national health insurance in 2000 and has followed them up ever since. LHID2000 was constructed by the government to provide a resource for research and is based on a representative sample of the whole population in Taiwan. The national health insurance covers more than 99% of the residents in Taiwan and provides comprehensive health care with a very low premium [37]. LHID2000 contained all the claim records from the beginning of the insurance program.

We identified all the live births from 1996 to 2000 in the LHID2000 and excluded those who had missing data on the insurance enrollment date or lived outside the Taiwan main island. The follow-up time was calculated as the duration from the enrollment date to the date of the first diagnosis of ASD or the end of the study period (31 December 2013).

### 2.2. Identification of Cases

The LHID2000 coded diagnoses according to the International Classification of Diseases, Ninth Revision, Clinical Modification (ICD-9-CM). We included three diagnosis codes as ASD: autistic disorder (ICD-9-CM code: 299.0), Asperger syndrome (299.8), and PDD-NOS (299.9) [36]. Individuals who had at least two identical target diagnoses on different dates were identified as having ASD. Because the National Health Insurance allows putting a tentative diagnosis on the insurance claim at the first visit for the illness and visiting more than one medical facility on the same day for the same illness, this inclusion criterion can exclude patients who were later diagnosed as having some diseases other than ASD. The date that the diagnosis code first appeared on a claim was defined as the date of diagnosis.

### 2.3. Exposure Assessment

The air pollutants studied included CO, NO_2_, O_3_, SO_2_, and PM_10_. We obtained the daily average value of each air pollutant from the hourly measurements made by the 69 automatic monitoring stations maintained by the Environmental Protection Administration on Taiwan Island from 1 January 1995 to 31 December 2000.

ArcMap GIS (Version 10.2.2, Environmental Systems Research Institute, Redlands, CA, USA) and its Geostatistical Analysts Extension were utilized to construct the estimates of ambient air pollution levels of each cohort member. The air pollution exposure gradients were computed by using the modified ordinary Kriging method [38], which can estimate the levels in unmeasured areas on the basis of data from the surrounding areas [39]. The adjusted spherical spatial model was used in this study to account for spatial autocorrelation [40]. The monitoring data were integrated into monthly data and interpolated to the township level.

The township level data were assigned to each cohort member by their postcodes in the database. In order to identify the critical prenatal exposure periods, we studied four exposure periods: the first trimester (preceding 7–9 month of birth), the second trimester (preceding 4–6 month of birth), the third trimester (preceding 1–3 month of birth), and gestation (the whole gestation period).

### 2.4. Covariates

According to the fact that males have a higher risk of developing ASD than females [15], we included sex in our analysis to evaluate and adjust for its effects. Likewise, because previous studies in Taiwan found an increasing trend in the occurrence of ASD [15], we included year of birth in our analysis to evaluate and adjust for the time trend. We also included comorbidities that are known to be related to ASD: anxiety (ICD-9-CM code: 300.0), bipolar disorder (296.4–296.8), depressive disorder (296.2, 296.3 and 311), intellectual disabilities (317–319), obsessive-compulsive disorder (300.3), phobic disorder (300.2), and preterm birth (765.xx).

### 2.5. Statistical Analysis

We used chi-square tests to evaluate differences in categorical variables and Student’s t tests to evaluate differences in continuous variables. We applied Cox proportional hazards models to evaluate associations between prenatal exposure to ambient air pollution and the risk of ASD. Multi-variate analyses were performed after univariate analyses. The air pollutants studied included CO, NO_2_, O_3_, SO_2_, and PM_10_. We used the mean level within the three-month period of each trimester as the indicator of the exposure in the analysis. For the whole gestation, we used the mean level during the whole gestation, estimated by taking the average of the mean levels of the three trimesters, as the indicator of the exposure. Due to confidentiality, the government did not provide data on the exact date of birth, just the year and month. The follow-up period ended on the date of the diagnosis, the date of leaving the insurance program (death or withdraw from the program), or the end of the study period, whichever came first.

In the multi-variate analyses, Cox proportional hazard models were used to adjust for the effect of other covariates. We first added sex and calendar year to the model (model 1), and then comorbidities were added (model 2). The effects of each pollutant on the risk of ASD were measured as an adjusted hazard ratio (HR) per ppm for CO, per 10 ppb for NO_2_, per 10 ppb for O_3_, per ppb for SO_2_, and per 10 μg/m^3^ for PM_10_, and the 95% confidence interval (95%CI) of each HR was constructed. The units were chosen using a previous study in Taiwan as the reference [36].

The two-tailed significance level (α level) was set at 0.05, and all the statistical analyses were performed using the SAS Enterprise Guide 6.1 for Windows (SAS Institute, Cary, NC, USA).

## 3. Results

### 3.1. Study Sample and Distribution of Patients

**Figure 1:**
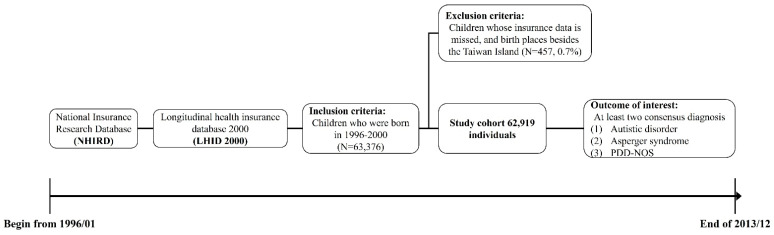
Flow chart of the study.

We included 63,376 individuals from the preliminary study cohort. After the exclusion of 52 individuals who had missing data on the insurance enrollment date and 405 who lived outside the Taiwan main island, the final study cohort included a total of 62,919 members (Figure 1). A total of 466 members were identified as having ASD (the ASD group) in the study cohort, yielding a cumulative incidence rate of 74.06 cases per 10,000 children in the study periods (Table 1). The median followed time of ASD patients was 5.66 years (mean = 6.77, standard deviation [SD] = 4.02 years), which means that 50% of the ASD patients were diagnosed at or before the age of 5.66 years old. Most members (82.83%) of the ASD group were males, and the ASD group had a higher male/female ratio than the non-ASD group (4.83 vs. 1.08, *p* < 0.001). The proportions of members with the comorbidities we studied were all higher in the ASD group than in the non-ASD group, and the most common comorbidity in the ASD group was intellectual disabilities (31.97%), followed by anxiety (14.16%).

**Figure 2:**
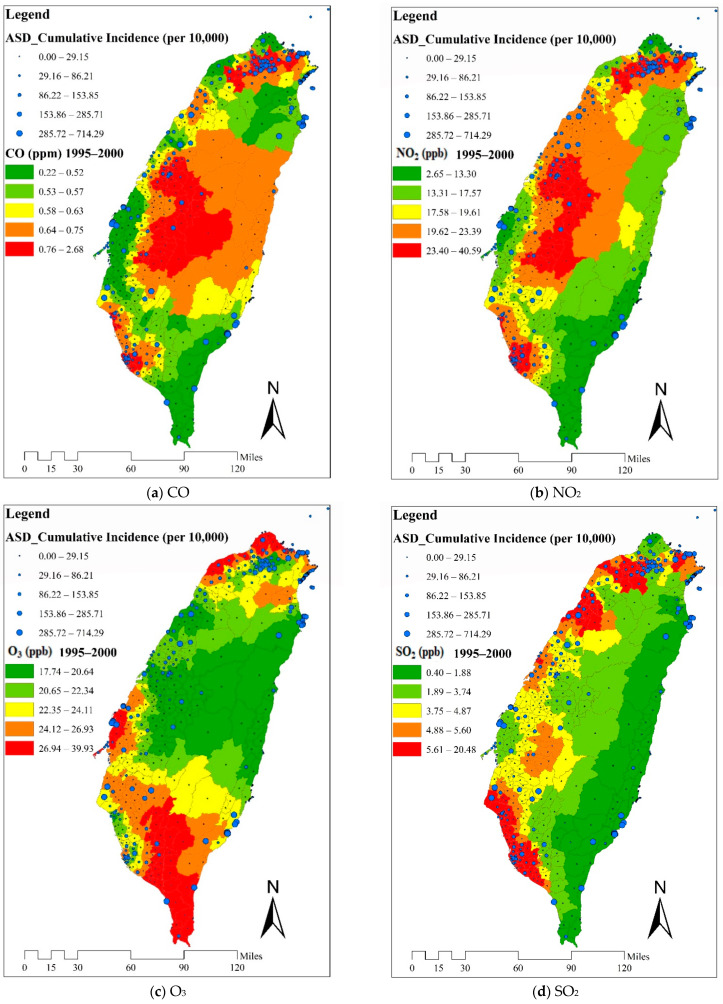

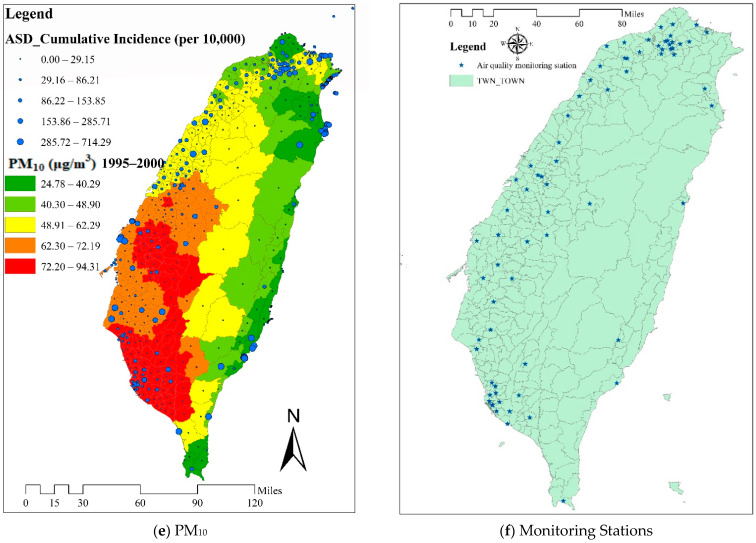
Cumulative incidence rates (per 10,000 children) of autism spectrum disorder and mean levels of air pollutants in Taiwanese townships during the study period.

The hot spots of children with ASD scattered around the main island of Taiwan without obvious geographical clustering (Figure 2). The distribution of hot areas of CO was similar to that of NO_2_ (Figure 2). A substantial proportion of the hot areas of O_3_ were in the southwest and northwest regions, but the distribution of hot areas of the SO_2_ was quite different from that of hot areas of O_3_. The hot areas of PM_10_ were clustered in the southwest region.

The text continues here (Figure 2 and Table 2).

### 3.2. Levels of Air Pollutants

**Figure 3:**
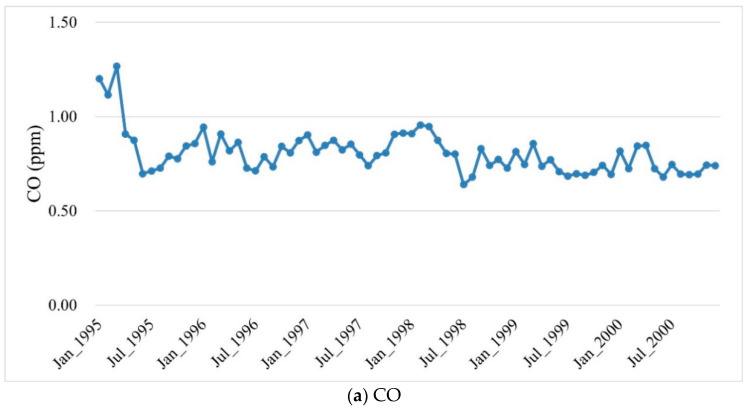

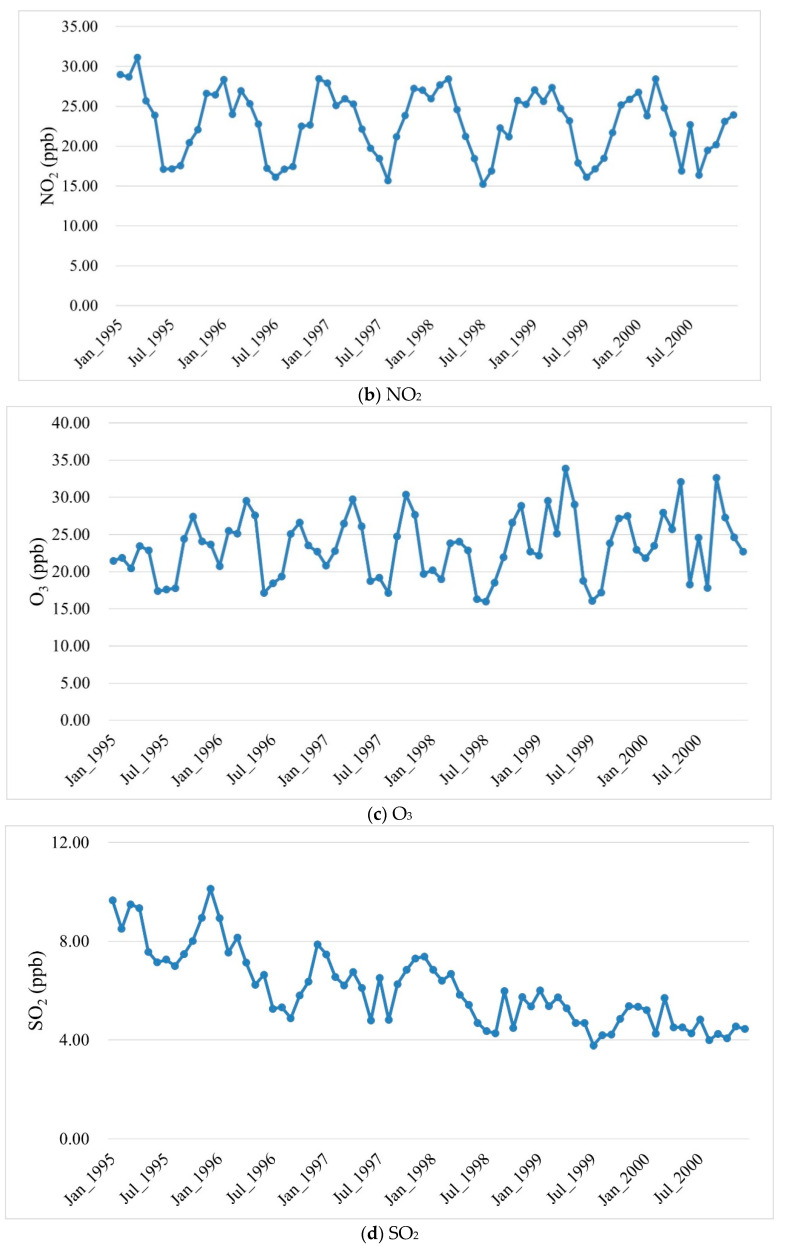

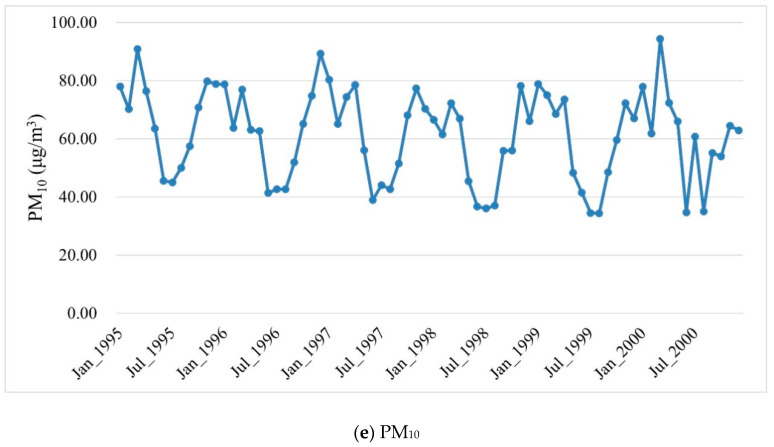
The monthly averages of air pollutant levels over the years of birth of the study cohort.

**Figure 4:**
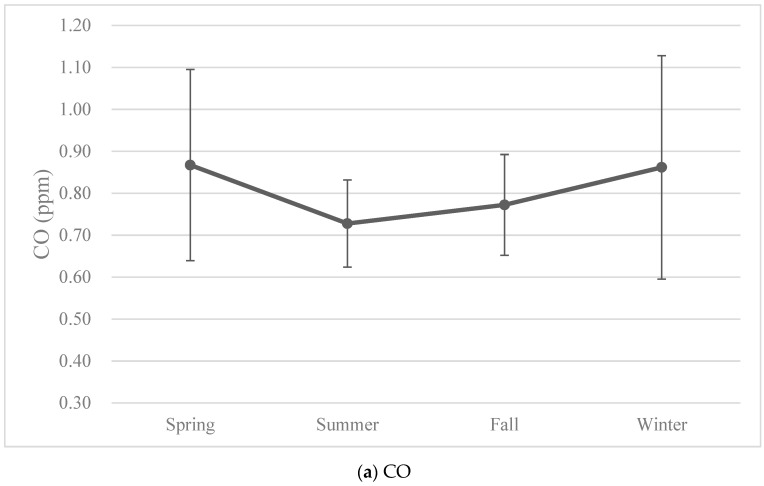

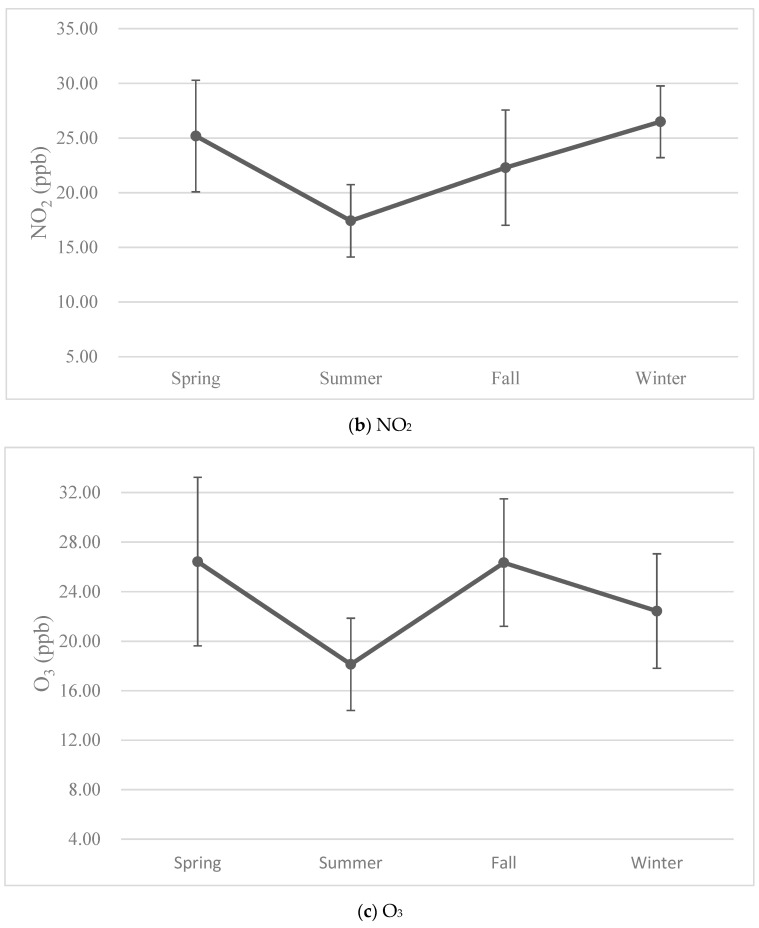

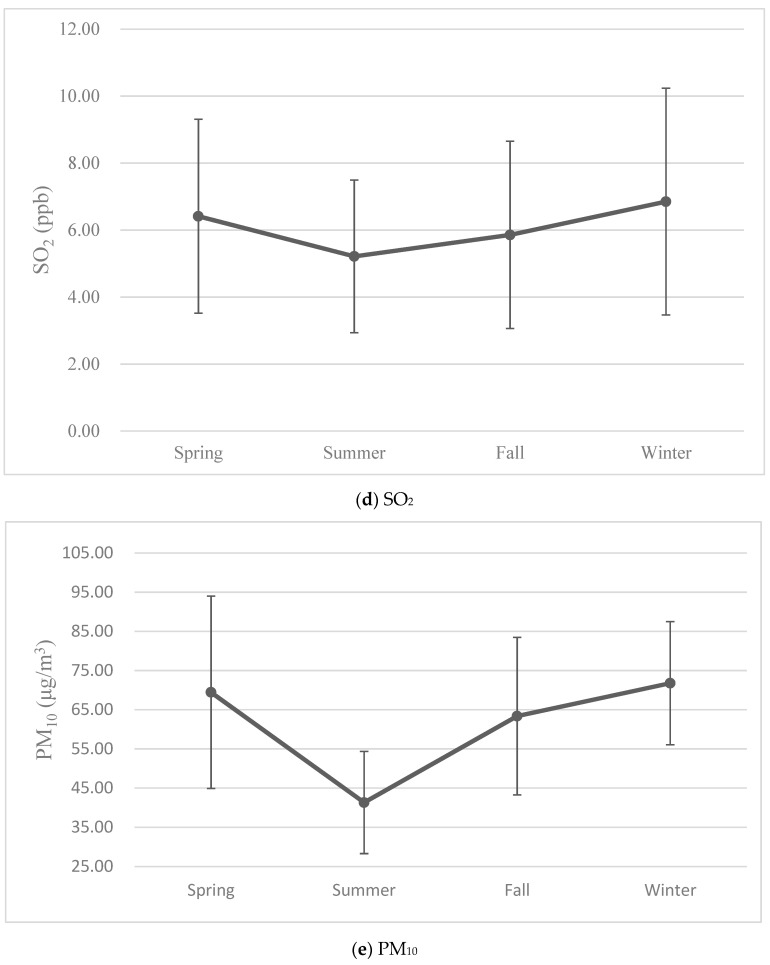
The seasonal variations of air pollutant levels over the years of birth of the study cohort. The data were presented as means with error bars indicating the range of ±1 standard deviation.

The monthly average levels of all air pollutants showed seasonal variations (Figure 3). In fact, most of the air pollutants had the same variation pattern, with the highest levels in winters and the lowest levels in summers, except for O_3_, which had the highest levels in falls (Figure 4).

### 3.3. Results from Univariate Analyses of Covariates

The risk of ASD was associated with several covariates we studied (Table 2). Males had a higher risk than females (hazard ratio [HR] = 4.45; 95%CI: 3.45–5.66). In addition, we observed an increasing trend with calendar year in the risk of ASD, with an HR of 1.12 (95%CI: 1.05–1.20) every year.

The proportions of members with the comorbidities we studied were all higher in the ASD group than in the non-ASD group. In particular, while there were 66 ASD patients having anxiety (14.16%), none of the members in the non-ASD group had the comorbidity. Other than that, intellectual disabilities were associated with the highest HR (34.26; 95%CI: 28.20–41.63), followed by obsessive compulsive disorder (HR = 25.98; 95%CI: 15.54–43.44).

### 3.4. Associations between Air Pollutants and Autism Spectrum Disorder

During gestation, the ASD group had significantly higher levels of CO and NO_2_ than the non-ASD group (Table 1). Yet, the levels of O_3_ and PM_10_ were significantly lower in the ASD group. The largest difference was observed in CO (more than 7% higher in the ASD group).

From Cox regression analyses, we found that the risk of ASD increased as the level of CO increased (Table 3). Specifically, the HR associated with 1 ppm increase in the gestation was 1.65 (95%CI: 1.32–2.07), and the association was also observed after adjusting for sex and calendar year (adjusted HR = 1.77; 95%CI: 1.41–2.22) and after further adjustment for comorbidities (adjusted HR = 1.88; 95%CI: 1.49–2.36). In all three trimesters, there was an association between CO levels and the risk of ASD. After adjusting for sex and calendar year, the associations were still statistically significant. With further adjustment for comorbidities, the adjusted HR associated with 1 ppm increase in the CO level decreased from 1.93 (95%CI: 1.55–2.39) in the first trimester to 1.75 (95%CI: 1.30–2.21) in the third trimester. We observed a similar pattern in the HRs associated with exposure to NO_2_. Specifically, the HR associated with each 10-ppb increase in the level of NO_2_ in gestation was 1.37 (95%CI: 1.18–1.60), and the association was also observed after adjusting for sex and calendar year (adjusted HR = 1.39; 95%CI: 1.19–1.62) and after further adjustment for comorbidities (adjusted HR = 1.42; 95%CI: 1.22–1.66) (Table 3). In all three trimesters, there was an association between NO_2_ levels and the risk of ASD. After adjusting for sex and calendar year, the associations were still statistically significant. With further adjustment for comorbidities, the adjusted HR associated with each 10-ppb increase in the level of NO_2_ decreased from 1.39 (95%CI: 1.22–1.58) in the first trimester to 1.18 (95%CI: 1.03–1.34) in the third trimester.

There were negative correlations of O_3_ and PM_10_ levels to the risk of ASD (Table 3). A 10-ppb increase in the level of O_3_ during gestation was associated with an HR of 0.73 (95%CI: 0.56–0.96). The association was also observed after adjusting for sex and calendar year (adjusted HR = 0.69; 95%CI: 0.52–0.91) and after further adjustment for comorbidities, and the adjusted HR was 0.74 (95%CI: 0.56–0.97) (Table 3). When the gestation was divided into trimesters, a negative correlation between the level of O_3_ and the risk of ASD was observed in all three trimesters, but none of the HRs reached statistical significance. After adjusting for sex and calendar year, the changes in the HRs were very small. With further adjustment for comorbidities, the adjusted HRs had very small changes and were very similar across the three trimesters, between 0.82 and 0.85 for each 10-ppb increase in the level of O_3_. The negative correlation between the level of PM_10_ and the risk of ASD was relatively stable. The HR associated with each 10 μg/m^3^ increase in PM_10_ during gestation was 0.86 (95%CI: 0.81–0.92), and the association was also observed after adjusting for sex and calendar year (adjusted HR = 0.87; 95%CI: 0.82–0.92) and after further adjustment for comorbidities (adjusted HR = 0.90; 95%CI: 0.85–0.96). After adjusting for sex and calendar year, the HRs generally decreased from the first to the third trimester, and an association was observed in all three trimesters, except that the HR associated with each 10 μg/m^3^ increase in the first trimester did not reach statistical significance (Table 3). After further adjustment for comorbidities, the adjusted HR in the first trimester was 0.98 (95%CI: 0.94–1.03), which decreased to 0.94 (95%CI: 0.90–0.99) in the second trimester and to 0.91 (95%CI: 0.87–0.96) in the third trimester.

No association between the level of SO_2_ and the risk of ASD was observed during gestation, or in any of the three trimesters (Table 3). The HRs associated with each 10-ppb increase in the level of SO_2_ were very close to the null value (differences no more than 0.02), before or after adjustment for other covariates.

## 4. Discussion

Previous studies on associations between prenatal exposures to air pollutants and childhood ASD are limited, especially those on specific exposure windows, and did not have consistent findings. Most of the studies were case-control studies conducted outside Asia. For example, a recent review of the literature retrieved 25 studies on this topic, and all but 3 were case-control studies, of which none were in Asia [41]. In this cohort study, we followed a representative sample of 62,919 newborns in Taiwan. We observed trimester-specific associations between levels of CO, NO_2_, and PM_10_ and the risk of childhood ASD after adjusting for other risk factors. In particular, levels of CO and NO_2_ in all three trimesters were associated with increased risks, and levels of PM_10_ in the second and third trimesters were associated with decreased risks.

### 4.1. Environmental Factors Contribute to Autism Spectrum Disorder

ASD was regarded as a genetic disease previously [3,22,42,43], but the concordance of monozygotic pairs was not fully explained by genetics [1]. Recent twin studies estimated that environmental factors contribute about as equally as genetic factors, and the reasons for this change may include the limitations in case numbers, inclusion criteria, and case definition in early studies [23,24].

The mechanisms through which environmental factors contribute to the development of ASD may include immune dysregulation, altered lipid metabolism, malnutrition, and higher oxidative stress. These conditions may influence the development of the brain in different stages, which include neuron cell differentiation, myelination, synaptogenesis, neural tube formation, and formation of the brain structure [44,45]. Air pollution might also affect neurodevelopment through indirect pathways, such as endocrine disruption, structural damage to brain tissues, and epigenetic changes [31,46,47,48,49]. Socioeconomic factors of the family may also contribute to the occurrence of ASD, but the findings in related studies were inconsistent [50,51]. Migration status has also been identified as a factor associated with ASD. A decreased risk of high-functioning autism was observed in children of migrant parents in Sweden [52], which is compatible to the finding of a lower incidence of developmental delays (including ASD) in children born to immigrant mothers in Taiwan [53]. Maternal factors may also affect the development of ASD, including age, nutritional status, health conditions, lifestyle, and breastfeeding [45,54,55,56,57,58,59,60,61].

### 4.2. Associations of Air Pollution and Autism Spectrum Disorder

There is growing literature on the association between ambient air pollution and ASD. However, the results of related studies were inconsistent, and even among studies that observed associations between ASD and air pollutants, the reported effect size varied across studies [31,33,36]. There was one study in Taiwan, but it was on postnatal exposure, not exposure during gestation [36]. Positive correlations of CO, O_3_, NO_2_, and SO_2_ with ASD were observed. Among studies that evaluated exposure in the prenatal period, a study found that ASD was associated with residential proximity to a freeway during all periods of gestation [31], and further analyses of the data indicated that exposure to traffic-related air pollution, such as NO_2_ and PM, during gestation was associated with ASD [33].

We observed positive correlations between the levels of CO and NO_2_ during gestation with the risk of ASD. The similar time trends observed in the HRs associated with CO and NO_2_ suggested that they associate with ASD through a similar mechanism. Studies have shown that exposures to CO and NO_2_ may introduce oxidative stress, which in turn may lead to immune dysregulation [62,63,64,65,66,67,68,69,70,71]. In addition, CO has higher affinity for binding hemoglobin than oxygen and leads to tissue hypoxia, which may generate partially reduced oxygen species in the brain after reoxygenation and thus damage neurons [72]. Likewise, exposure to NO_2_ may introduce reactive oxygen species (ROS) in the neuronal cells, which can mediate apoptosis of the cells [73]. Moreover, fetal hemoglobin binds to CO stronger than maternal hemoglobin, which leads to hypoxia and reoxygenation in fetal tissues [74,75,76,77]. NO_2_ can cause inflammation of the lung tissue after inhalation and may result in systemic inflammation, including inflammation in the placenta [78,79,80]. The inflammation may lead to the generation of ROS, which may damage neurons in the brain through the same mechanism as mentioned above. In addition to inducing systemic inflammation, air pollutants in maternal blood may pass through the placenta to the fetus in experimental animals and cause local inflammation in the brain [46], which may also lead to the generation of ROS and thus damage the brain. Animal studies have also shown that inhalation of NO_2_ may cause damages in the hippocampus and depletion of lipids in the cerebral hemispheres, cerebellum, and mid brain [81,82,83].

In many regions around the world, motor vehicle exhaust is the main source of CO and NO_2_, and it has been shown that exposure to traffic-related air pollution may lead to endothelial dysfunction, breakdown of the blood-brain-barrier, microglia activation, and effects on the dopaminergic system [84,85,86,87,88]. Nonetheless, some researchers believe that NO_2_ is just a surrogate of air pollution rather than a direct source of neuroinflammation [89]. For example, it might represent the neighborhood socioeconomic status [90], which is associated with ASD. Because the association can be positive or negative depending on the region under study, this could be a reason for inconsistent findings on the correlation between NO_2_ and ASD in different regions. In addition, there is also evidence that chronic exposure to noise may be associated with decreased cognitive function in children [91], and the main source of NO_2_ in many regions in the world is traffic, which is also a main source of noise.

We observed negative correlations between the O_3_ level during gestation and the risk of developing childhood ASD, which is compatible with a study in Taiwan that observed increases in neurodevelopment scores at 6 and 18 months of age associated with the O_3_ levels in both the second and the third trimesters [92]. A recent meta-analysis of four studies did not observe a significant association between prenatal exposure to O_3_ and the risk of ASD [41], but all the four studies were conducted in the U.S.A. More studies outside the U.S.A. are needed to clarify this issue. A possible reason for observing a negative association if there are in fact no effects is the occurrence of live birth bias, which is introduced when only live births are studied. In other words, if O_3_ decreased the survival of fetuses with ASD, less such fetuses could survive till birth and thus led to underestimation of the risk, and even a negative association. However, this is not likely to be the case in our study because it should also be observed in studies around the world, not just in Taiwan.

We also observed negative correlations between the PM_10_ level during gestation and the risk of developing childhood ASD. While this is inconsistent with findings in previous studies in California [32,33], it is compatible with a study in Denmark, which observed an adjusted odds ratio of 0.95 (95%CI: 0.91–1.00) for each interquartile range (IQR: 3.80 μg/m^3^) [93]. It is also in line with a collaborative study of four European population-based birth/child cohorts, which found a negative association between PM_10_ levels during gestation and autistic traits, and the adjusted odds ratio associated with each 10 μg/m^3^ increase of PM_10_ was very close to our finding (0.90 vs. 0.903) but did not reach statistical significance [94]. The strongest association was observed in the Spain cohort, with an odds ratio of 0.40 (95%CI: 0.04–3.91). In fact, a systematic review that identified 13 studies on the association between PM exposure and ASD found 4 of them did not observe an association while 8 observed positive associations restricted to specific exposure windows, which did not reach statistical significance [95]. Another systematic review that identified 14 studies found low external consistency in results among studies on PM and ASD, even among the 4 specifically on diesel PM and ASD [96]. Furthermore, a recent meta-analysis of nine studies did not observe a significant association between prenatal exposure to PM_10_ and the risk of ASD using three different methods, and considerable heterogeneity among the studies was found [41]. The inconsistency among studies may be due to several reasons. Most importantly, PM_10_ is a complex mixture simply classified by size, and the composition varies by place, and even varies by time in the same place, depending on the sources. While transportation is a main source in many places, such as in California, emission by coal-burning power plants is a relatively important source in Taiwan. This may explain why the effects of PM appear to be similar to NO_2_ in California but not in Taiwan. In addition, the PM_10_ level varies across studies. For example, the mean level of PM_10_ in the California study was 26–36 μg/m^3^, but it was 41–75 μg/m^3^ in our study. Furthermore, as in the case of NO_2_, transportation is the main source of PM_10_ in many regions in the world, and thus PM_10_ may represent the neighborhood socioeconomic status [90].

### 4.3. Trimester-Specific Associations

We observed trimester-specific associations of CO, NO_2_, and PM_10_ levels with the risk of childhood ASD. In addition, we found the positive correlations of CO and NO_2_ levels with ASD decreased with time while the negative correlation between the PM_10_ level and ASD increased with time. These findings are rarely reported in the literature. In a study in California, the adjusted odds ratios associated with an increase of 2 standard deviations from the mean value (14.1 ppb) in the level of NO_2_ were 1.44, 1.61, and 1.64, respectively, for the first, second, and third trimester, which is compatible with our observation, but CO was not included in the study [32,33]. In fact, using “carbon monoxide” and “autism” as keywords to search literature in PubMed, we failed to find any research articles on the association between prenatal exposure to CO and ASD.

The first trimester is a critical ontogenetic period in the brain, which is a major part of the neuronal cell formation, neuronal migration, and neuronal maturation [97,98,99,100,101]. From the 1st to the 20th gestational week, the neurogenesis of the brain occurs, and the neural tube starts to develop in the 5th gestational week and completes the development shortly [102]. Likewise, the greater part of the neuroblast is generated from the 5th week till the 25th gestational week [103]. The neuronal cells migrate once they are formed. The peak of the neuronal migration is between the 12th and 16th gestational week, and the migration stops around the 30th gestational week [104,105,106]. The neuronal cell maturation also ends in the early phase, which is around the 24th gestational week [97,100]. Therefore, the majority of the brain formation occurs during the first half of gestation [107,108]. In our study, exposures to the two air pollutants that were found to have positive correlations with ASD in the first trimester were associated with the largest HR, and the HR decreased with time, which indicated the associations are biologically plausible.

### 4.4. Strengths and Limitations of the Study

Studies on the association between prenatal exposure to air pollution and ASD outside the U.S.A. are limited, and such studies were rarely conducted in Asia. The current study is most likely the first one to report trimester-specific risk estimates between prenatal exposure to air pollutants and the risk of ASD in an Asian population.

Besides the features mentioned above, the current study has several strengths. First of all, we used a large population-based cohort to conduct the study. The size of the cohort allowed us to study conditions that are relatively rare and adjust for the effects of many potential confounders at the same time. In addition, the study cohort was a random sample of the enrollees of the National Health Insurance, which included over 99% of the residents in Taiwan, and the effects of selection bias were thus minimized. The diagnosis of ASD requires expertise and takes time and resources. In our study, the diagnosis was determined as confirmed when it was listed at least in two different dates, which reduced the misclassification of outcomes. Furthermore, we used the data from air quality monitoring stations operated by the government, and therefore the measurements were standardized and reliable.

A major limitation of our study is that the LHID2000 does not have information on the mother, and therefore we were unable to adjust for maternal factors, such as age, nutrition status, family history, medical condition, substance abuse, etc. In addition, we did not conduct actual personalized measurements of the air pollutants. Instead, we used the Kriging method to estimate the levels in unmeasured areas on the basis of data from the surrounding areas and construct the estimates of ambient air pollution levels of each cohort member, which would introduce errors. However, this approach has been used by all the previous large-scale studies, and even many studies that had much smaller samples. Besides, there are tradeoffs from using personal exposure measures vs. using proxy measures (Weisskopf and Webster, 2017). It is possible that some of the pregnant women did not live at the address they registered. However, because they did not know whether their children would develop ASD during the study period, the errors were most likely to occur randomly, and so misclassification bias is unlikely to be introduced in our study. There are other environmental factors that might contribute to the development of ASD, including other air pollutants, such as ultrafine particles, but we did not have the data to take them into account.

Using exposure data on the basis of residence, we assumed that exposure was mostly limited to the residence at the time and that the residence did not change during the given period. These are assumptions commonly used in most similar studies in the literature. For example, while no studies have been conducted on prenatal exposure in Taiwan, one study was conducted on postnatal exposure [36]. This study used exposure data on the residence and assumed the residence did not change over a period up to four years, but our study assumed that the residence did not change over a period of up to nine months only, which is more likely to hold.

In our study, levels of the pollutants were highly correlated, particularly the three pollutants found to be associated with ASD (CO, NO_2_, and PM_10_, with Spearman correlation coefficients of at least 0.82). Therefore, we were unable to adjust for other air pollutants using two-pollutant models as in the previous study by Jung et al. on postnatal exposure in Taiwan [36], because putting them in the same model would violate the main assumption of independence between the two pollutants. However, in the study by Jung et al., the risk estimates did not have differences between single-pollutant and two-pollutant models that were remarkable enough to change the conclusions, except that PM_10_ did not have a significant association with ASD in the single-pollutant model but had a significant negative association with ASD in the PM_10_+NO_2_ model, which was consistent with the finding in our study. They observed significant positive associations with CO and NO_2_ as in our study but also significant positive associations with O_3_ and SO_2_, suggesting that O_3_ and SO_2_ might have different effects before and after birth.

## 5. Conclusions

We found the levels of CO and NO_2_ in all three trimesters were associated with an increased risk of developing childhood ASD in Taiwan, but exposure to O_3_, SO_2_, or PM_10_ did not appear to be risk factors. The risks associated with CO and NO_2_ were most prominent in the first trimester and decreased with time. These findings indicate that prenatal exposure to environmental pollution may contribute to the development of ASD. Some of the findings are different from those observed in the U.S.A. and some European countries. As the mix of pollutants, and even the level of the same pollutant, may vary across geographic regions, it may not be unexpected that pollutants show different associations. In fact, a recent systematic review identified 25 studies and concluded that “patterns in associations over trimesters were inconsistent among studies and among air pollutants”. [41]. Nonetheless, further studies, particularly those using personal air sampling and those in the East Asia region, are warranted to confirm our findings. Different modeling approaches, such as distributed lag models, might help describe the time-specific effects in greater details. In addition, further studies should be conducted to evaluate the associations between CO and ASD, which are rarely reported in the literature.

## Figures and Tables

**Table 1:** Comparison between cohort members with (ASD) and without (non-ASD) autism spectrum disorder.

	N (%) ^1^		
Variables	ASD (N = 466)	Non-ASD (N = 62,453)	*p*-Value
Sex (male/female ratio)	4.83	1.08	<0.001
Male	386 (82.83)	32,427 (51.92)	
Female	80 (17.17)	30,016 (47.72)	
Birth year			0.026
1996	78 (16.74)	13,377 (21.42)	
1997	90 (19.31)	13,538 (21.68)	
1998	101 (21.67)	11,343 (18.16)	
1999	101 (21.67)	11,809 (18.91)	
2000	96 (20.60)	12,386 (19.83)	
Followed year (mean ± SD ^1^)	6.76 ± 4.02	15.30 ± 2.20	
Comorbidity			
Bipolar disorder	9 (1.93)	61 (0.10)	<0.001
Depressive disorder	15 (3.22)	195 (0.31)	<0.001
Anxiety	66 (14.16)	0 (0.00)	<0.001
Phobic disorder	3 (0.64)	60 (0.10)	0.012
Obsessive compulsive disorder	15 (3.22)	70 (0.11)	<0.001
Intellectual disabilities	149 (31.97)	777 (1.24)	<0.001
Preterm birth	10 (2.15)	514 (0.82)	0.006
Air pollutant level (mean ± SD)			
CO (ppm)	0.89 ± 0.36	0.83 ± 0.32	<0.001
NO_2_ (ppb)	25.47 ± 6.12	24.34 ± 6.01	<0.001
O_3_ (ppb)	21.60 ± 3.61	21.99 ± 3.54	0.019
SO_2_ (ppb)	5.76 ± 3.29	5.80 ± 3.47	0.783
PM_10_ (μg/m^3^)	56.83 ± 15.24	60.27 ± 16.20	<0.001

^1^ Column percentage, including members with missing data. Abbreviations: SD = standard deviation.

**Table 2:** The hazard ratio and its 95% confidence interval (95%CI) associated with each covariate for autism spectrum disorder.

Covariate	Hazard Ratio	(95% CI)
Male	4.45	(3.50–5.66) *
Calendar year	1.12	(1.05–1.20) *
Comorbidity		
Bipolar disorder	17.66	(9.13–34.14) *
Depressive disorder	9.68	(5.79–16.18) *
Phobic disorder	6.43	(2.07–20.03) *
Obsessive compulsive disorder	25.98	(15.54–43.44) *
Intellectual disabilities	34.26	(28.20–41.63) *
Preterm	2.77	(1.48–5.19) *

* *p* value < 0.05.

**Table 3:** Crude and adjusted hazard ratios for autism spectrum disorder associated with air pollutants.

	Crude Hazard Ratio (95%CI)	Adjusted Hazard Ratio (95%CI)	
		Model 1 ^1^	Model 2 ^2^
CO (ppm)			
Gestation	1.65 (1.32–2.07) *	1.77 (1.41–2.22) *	1.88 (1.49–2.36) *
1st Trimester	1.68 (1.36–2.09) *	1.79 (1.44–2.23) *	1.93 (1.55–2.39) *
2nd Trimester	1.56 (1.25–1.96) *	1.66 (1.33–2.09) *	1.77 (1.41–2.22) *
3rd Trimester	1.57 (1.25–1.97) *	1.68 (1.34–2.11) *	1.75 (1.39–2.21) *
NO_2_ (10 ppb)			
Gestation	1.37 (1.18–1.60) *	1.39 (1.19–1.62) *	1.42 (1.22–1.66) *
1st Trimester	1.32 (1.17–1.50) *	1.34 (1.19–1.52) *	1.39 (1.22–1.58) *
2nd Trimester	1.22 (1.07–1.38) *	1.22 (1.07–1.38) *	1.25 (1.10–1.42) *
3rd Trimester	1.17 (1.04–1.33) *	1.18 (1.04–1.34) *	1.18 (1.03–1.34) *
O_3_ (10 ppb)			
Gestation	0.73 (0.56–0.96) *	0.69 (0.52–0.91) *	0.74 (0.56–0.97) *
1st Trimester	0.83 (0.68–1.02)	0.81 (0.66–0.99) *	0.85 (0.69–1.04)
2nd Trimester	0.82 (0.67–1.00)	0.79 (0.64–0.97) *	0.82 (0.67–1.02)
3rd Trimester	0.86 (0.70–1.05)	0.83 (0.67–1.01)	0.84 (0.68–1.03)
SO_2_ (ppb)			
Gestation	0.99 (0.97–1.02)	1.00 (0.98–1.03)	1.01 (0.99–1.04)
1st Trimester	1.00 (0.98–1.02)	1.01 (0.99–1.03)	1.02 (1.00–1.04)
2nd Trimester	0.99 (0.97–1.02)	1.00 (0.98–1.03)	1.01 (0.98–1.03)
3rd Trimester	0.99 (0.96–1.01)	1.00 (0.97–1.02)	1.00 (0.98–1.03)
PM_10_ (10 μg/m^3^)			
Gestation	0.86 (0.81–0.92) *	0.87 (0.82–0.92) *	0.90 (0.85–0.96) *
1st Trimester	0.95 (0.91–0.99) *	0.96 (0.92–1.00)	0.98 (0.94–1.03)
2nd Trimester	0.92 (0.89–0.96) *	0.92 (0.88–0.96) *	0.94 (0.90–0.99) *
3rd Trimester	0.90 (0.86–0.94) *	0.90 (0.86–0.95) *	0.91 (0.87–0.96) *

^1^ Adjusted for sex and calendar year. ^2^ Adjusted for sex, calendar year, and comorbidities. Abbreviation: CI = confidence interval. * *p* value < 0.05.

## Data Availability

All the data used in this study are publicly available from the Taiwanese government upon approval of application and payment of fees.
